
JOURNAL INFORMATION
==============================
NLM Title Abbreviation: ScientificWorldJournal
Journal ID: ScientificWorldJournal
NLM Journal ID: 101131163
Journal ID: TSWJ

The Scientific World Journal

ISSN: 2356-6140
EISSN: 1537-744X
Publisher: Wiley

ARTICLE INFORMATION
==============================
PMCID: PMC6084091
PMID: 30147491
DOI: 10.1100/tsw.2001.147
Article ID: 976031
Article version: 1
Subjects: Short Report

Transduction of TAT/PTD Antiapoptotic Fusion Proteins in Pancreatic Islets

Embury Jennifer
Ribeiro Melina
Klein Dagmar
Pileggi Antonello
Molano R. Damaris
Fraker Christopher
Kenyon Norma
Ricordi Camillo
Inverardi Luca
Pastori Ricardo L. *
Diabetes Research Institute, University of Miami School of Medicine, Miami, FL 33136, USA
*Ricardo L. Pastori: rpastori@med.miami.edu
Publication date: 2001
Electronic publication date: 2001 Nov 7
Volume: 1
First page: 41
Last page: 41
Copyright: Copyright © 2001 Jennifer Embury et al.
Copyright year: 2001
License: This is an open access article distributed under the Creative Commons Attribution License, which permits unrestricted use, distribution, and reproduction in any medium, provided the original work is properly cited.
License URL: https://creativecommons.org/licenses/by/3.0/

==============================
Miami *Nature Biotechnology* Short Reports
*TheScientificWorld* (2001) 1(S3), 41SR
ISSN 1532-2246; DOI10.1100/tsw.2001.147

### TRANSDUCTION OF TAT/PTD ANTIAPOPTOTIC FUSION PROTEINS IN PANCREATIC ISLETS

Jennifer Embury, Melina Ribeiro, Dagmar Klein, Antonello Pileggi, R. Damaris Molano, Christopher Fraker, Norma Kenyon, Camillo Ricordi, Luca Inverardi, and *Ricardo L. Pastori

Diabetes Research Institute, University of Miami School of Medicine, Miami, FL 33136, USA
*rpastori@med.miami.edu

**INTRODUCTION.** With the development of new strategies to avoid immunological rejection, transplantation of pancreatic islets has become a therapeutic reality to cure diabetes (1-2). However, despite the progress in islet isolation procedures, a single donor transplant does not provide enough islets to attain insulin independence. There is evidence that significant loss of islet cells takes place during isolation due to the triggering of apoptosis (3). There is also substantial evidence that reduction of isolation-induced apoptosis can improve the success rates of islet transplantation. The goal of this proposal is to address the needs for reduced apoptosis and improved viability of islets in conjunction with islet isolation procedures. We present in this study novel transduction methods that allow manipulation of islets to reduce apoptosis. Proteins can be directly transferred to cells when they are linked to protein transduction domains (PTDs), small peptide domains that can freely cross cell membranes. In particular, proteins fused to an 11-amino acid PTD from the human immunodeficiency virus, the TAT protein, readily diffuse across membranes and are efficiently transduced into virtually any cell type (4). The expression as well as the biological function of the TAT fusion protein are temporary without permanent modification of the cell sensitivity to apoptosis, thus avoiding undesirable long-term effects.

**METHODS.** *Cloning and related techniques* The recombinant TAT antiapoptotic fusion proteins were generated by subcloning the coding region cDNAs in frame with the TAT leader peptide using the pTAT bacterial expression vector, generously provided by Steven Dowdy from Washington University School of Medicine, St Louis. A 6xHis-affinity tag allowed the purification of the fusion protein through affinity chromatography.

**RESULTS.**

- βgalactosidase fused to the 11-amino acid that constitute the PTD of the HIV/TAT protein, allowed protein transduction to pancreatic islets in a time and dose dependent mode with an efficiency of 80 % of islet cells.
- TAT fusion proteins do not affect the insulin secretion capability of islets as assessed by glucose static incubation and by reversion of hyperglycemia in diabetic nude mice.
- In additional experiments, a TAT/PTD antiapoptotic fusion protein prevent apoptosis induced by different apoptotic stimuli when transduced to islets, probing that TAT/PTD proteins are biologically active within pancreatic islets.

**DISCUSSION.** The TAT/PTD fusion protein delivery system has a tremendous potential in improving the current islet isolation procedure. TAT fusion proteins are delivered efficiently to islets and pancreatic tissue, without impairing islet function [our preliminary results]. It is also a distinct advantage of the TAT/PTD system that transduced proteins present the cells only transiently. It is clear that improving islet viability is of utmost importance for the successful outcome of islet transplantation. Prevention of islet apoptosis due to isolation stress may allow for a greater number of healthy functional islets per isolation making it possible to perform islet transplantation with one donor pancreas per one or more recipients. This would significantly reduce transplantation wait times, which now reflect the critical shortage of organs. The potential that one donor pancreas may consistently provide islets for one recipient or more would be a major advance in clinical transplantation.

**REFERENCES.**

1. Hering, B.J. and Ricordi, C. (1999) Graft 2, 12-15
2. Shapiro, A.M., Lakey, J.R., Ryan, E.A., Korbutt, G.S., Toth, E., Warnock, G.L., Kneteman, N.M., and Rajotte, R.V. (2000) N. Engl. J. Med. 343, 230-238
3. Cattan, P., Berney, T., Schena, S., Molano, D., Pileggi, A., Vizzardelli, C., Ricordi, C., and Inverardi, L. (2000) Transplantation (in press)
4. Schwarze, S.R., Ho, A., Vocero-Akbani, A., and Dowdy, S.F. (1999) Science 285, 1569-1572
